# A Machine Vision-Based Method for Monitoring Scene-Interactive Behaviors of Dairy Calf

**DOI:** 10.3390/ani10020190

**Published:** 2020-01-22

**Authors:** Yangyang Guo, Dongjian He, Lilong Chai

**Affiliations:** 1 College of Mechanical and Electronic Engineering, Northwest A&F University, Yangling 712100, China; yangyang.guo@uga.edu; 2 Key Laboratory of Agricultural Internet of Things, Ministry of Agriculture and Rural Affairs, Yangling 712100, China; 3 Shaanxi Key Laboratory of Agricultural Information Perception and Intelligent Service, Yangling 712100, China; 4 Department of Poultry Science, College of Agricultural and Environmental Sciences, University of Georgia, Athens, GA 30602, USA

**Keywords:** dairy calf, animal behaviors, computer vision, scene-interaction

## Abstract

**Simple Summary:**

Requirements for dairy products are increasing gradually in emerging economic bodies such as China, so it is critical to monitor and maintain the health and welfare of the increasing population of dairy cattle, especially dairy calves (over 20% mortality). In this study, a new method was built by combining background-subtraction and inter-frame difference methods to monitor the behaviors of dairy calf. By using the new model and motion characteristics of the calf in different areas of the enclosure, the scene-interactive behaviors of entering or leaving the resting area, turning around, and stationary (no movement) were identified automatically with a 93–97% success rate. This newly developed method provides a basis for inventing evaluation tools to monitor calves’ health and welfare on dairy farms.

**Abstract:**

Requirements for animal and dairy products are increasing gradually in emerging economic bodies. However, it is critical and challenging to maintain the health and welfare of the increasing population of dairy cattle, especially the dairy calf (up to 20% mortality in China). Animal behaviors reflect considerable information and are used to estimate animal health and welfare. In recent years, machine vision-based methods have been applied to monitor animal behaviors worldwide. Collected image or video information containing animal behaviors can be analyzed with computer languages to estimate animal welfare or health indicators. In this proposed study, a new deep learning method (i.e., an integration of background-subtraction and inter-frame difference) was developed for automatically recognizing dairy calf scene-interactive behaviors (e.g., entering or leaving the resting area, and stationary and turning behaviors in the inlet and outlet area of the resting area) based on computer vision-based technology. Results show that the recognition success rates for the calf’s science-interactive behaviors of pen entering, pen leaving, staying (standing or laying static behavior), and turning were 94.38%, 92.86%, 96.85%, and 93.51%, respectively. The recognition success rates for feeding and drinking were 79.69% and 81.73%, respectively. This newly developed method provides a basis for inventing evaluation tools to monitor calves’ health and welfare on dairy farms.

## 1. Introduction

The global population is predicted to reach 9.5 billion in 2050, then the requirement for the animal protein (e.g., eggs, meat, and milk) is expected to increase by over 70% in 2050 as compared to 2005 [1]. Providing food for the increasing world population with limited natural resources is a grand challenge for animal agriculture. Requirements for dairy products (e.g., milk) are increasing gradually in emerging economic bodies such as China. However, it becomes critical and challenging to maintain the health and welfare of the increasing population of dairy cattle, as the combined mortality rate of dairy calves and replacement heifers in Chinese Holstein cattle was about 21% [2].

In recent years, animal imaging analysis or phenotyping has been tested to monitor animal behavior and health on dairy farms [3,4,5,6,7]. The behaviors of dairy cattle reflect their physiological and welfare conditions, and thus can be applied to improve our understanding of farm animal production, health, and welfare [8]. Traditionally, direct contact method (i.e., attaching sensors to animal body directly) has been the primary form of animal welfare monitoring [9,10,11,12], which may affect animal welfare or health over time. With the enhancement in machine learning technology, images or video information can be analyzed to recognize and classify specific animal behaviors [13,14,15,16,17,18,19].

Progress was made in analyzing animal scene-interactions behaviors such as feeding, drinking, and locomotion [20,21,22]. By analyzing image/video and scene information of the drinking area, the location and drinking behavior of cows was characterized [23]. Based on the top view of a bull barn, Meunier [24] divided the barn layout into eating, walkway, resting, and milking areas. Positional information for dairy cows was obtained with a real-time location system, which generated the real-time behavioral status of cows, and the time period of each individual behavior such as feeding and resting, and interactive behaviors. The behaviors of individual and group calves are different from dairy cows in many ways due to changes in body size, feeding/drinking, and locomotion, etc., which guaranty the study in devising a new monitoring system. As about 50%–60% of dairy production mortality occurs at the calf stage, calves’ management determines the general economic performance of a dairy farm [25,26]. Therefore, monitoring the interactive behaviors of dairy calves in their living scene (e.g., calf pen) will provide health and welfare information for producers to improve the early stage management of on-farm dairy production.

The objectives of this study were to (1) improve the background model development for analyzing dairy calf behaviors based on collected image/video information in a scene/pen; and (2) test the newly developed method for recognizing dairy calf’s behaviors of entering/leaving, rest, drinking/feeding in pen.

## 2. Materials and Methods

### 2.1. Experimental Setup and Image Collection

**Figure 1:**
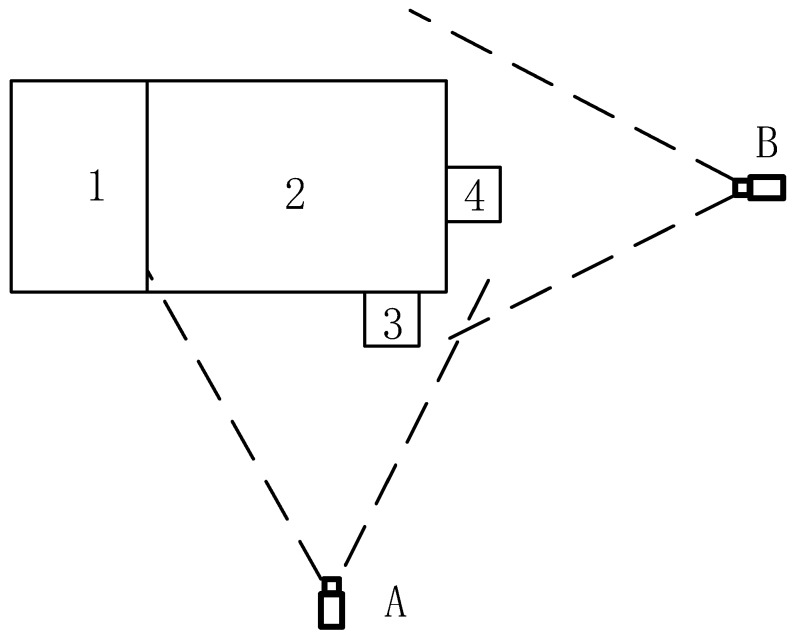
Video collection setup. **1**. Rest area; **2**. Activity area; **3**. Feeding-basin; **4**. Drinking-basin. **A**. Front-view camera; **B**. Side-view camera.

**Figure 2:**
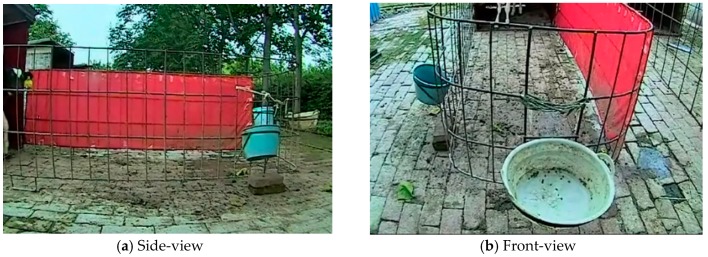
Test site. Red canvas was used to reduce background interference to side-view.

Two cameras were set up (DS-2CD4012, Hikvision, Hangzhou, China) for video/image collection on a commercial dairy farm (Keyuan Clone Ltd., Yangling, China) for monitoring a two-month old Holstein dairy calf in a rectangular fenced enclosure (4 × 2 × 1.5 m). Experimental setup is shown in Figure 1. The Camera A on the length side of the fence monitored the calf activity from the side with a wide angle of view. The vertical height of Camera A was the half height of fence (i.e., 0.75 m), and the horizontal distance to the fence was set to cover the whole activity area of the calf. Camera B was positioned at a height of 1.8 m on the short side of the fence, inclined slightly downwards. Calf’s eating and drinking behaviors were monitored, as shown in Figure 1 and Figure 2. Image/video data were collected from 07:00 to 18:00 h each day in July 2013. A single video file was generated per day. The video was captured at 25 frames/s, 2000 kb/s, and with a resolution of 704 pixels (horizontal) × 576 pixels (vertical) (in PAL format). The data-processing computer consisted of a CPU (Intel Core I5-2400, 3.2 GHz) with 8 GB memory and a 500 GB hard disk. Sample data were read and processed using MATLAB 2014b.

### 2.2. Calf-Target Detection Method

Common target-detection methods include the inter-frame difference [27], the background-subtraction [28], the Gaussian Mixture Model [29], and the ViBe [30], etc. The Gaussian Mixed Model and ViBe methods have been used to detect moving targets, but were not efficient enough in monitoring the stationary status of animals. The background-subtraction method is able to detect stationary targets, but is susceptible to background interference. The inter-frame difference method has stronger anti-interference properties but cannot detect stationary targets. In this study, the background-subtraction and inter-frame difference methods were integrated to rebuild the background model for individual calf detection. Images processing steps include:

(1) Median filtering was performed on the video frames; RGB images were converted to grayscale images, then the background frame was selected, background subtraction was performed, and small areas were removed;

(2) Otsu’s method was used to segment the image. A square 4 × 4 pixel element was selected for the closing operation and hole filling;

(3) The top, bottom, left, and right borders of the non-zero region were expanded outward by five pixels to obtain new borders of a search box that contained as much as possible of the target region. If the border of the search box overlapped with the image border, the image border was considered to be the border of the search box

(1) $$\left\{ \begin{array}{c} U_{end}=U_{test}-5,\;if\;U_{test}-5\leq 0,U_{end}=0\\ D_{end}=D_{test}+5,\;if\;D_{test}+5\leq 576,D_{end}=576\\ L_{end}=L_{test}-5,\;if\;L_{test}-5\leq 0,L_{end}=0\\ R_{end}=R_{test}+5,\;if\;R_{test}+5\leq 704,R_{end}=704 \end{array}\right.$$

where $U_{end}$ is the top boundary of the target area, $D_{end}$ is the bottom of the target area, $L_{end}$ is the left boundary of the target area, $R_{end}$ is the right boundary of the target area, $U_{test}$ is the top boundary of the non-zero area, $D_{test}$ is the bottom of the non-zero area, $L_{test}$ is the left boundary of the non-zero area, and $R_{test}$ is the right edge of the non-zero area.

**Figure 3:**
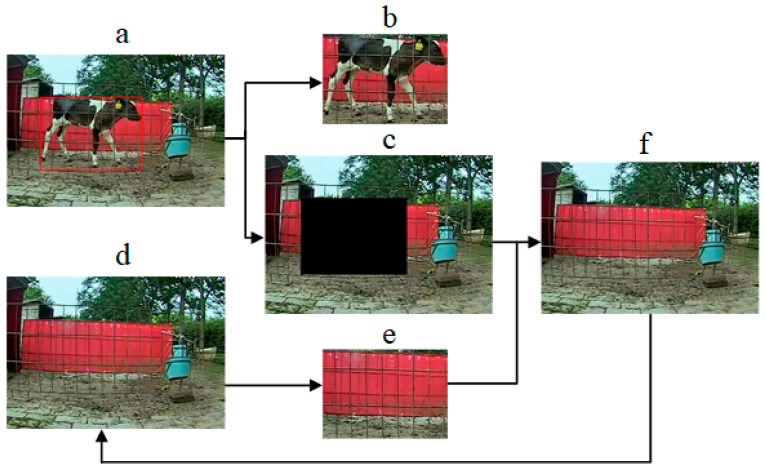
Model of background updating: **a**. target test results and the red box represents target area; **b**. target area; **c**. background area after removing target area; **d**. previous synthetic background frame; **e**. background area corresponding to target area; and **f**. synthetic background frame.

(4) Using the above steps, the target area was detected and proposed, as shown in Figure 3b, and the parts outside the target area were extracted (Figure 3c). The region corresponding to the target region in the previously synthesized background frame (Figure 3d) was extracted (Figure 3e), and a new background frame was synthesized with Figure 3c as the new background image for the next target-detection frame (Figure 3f).

### 2.3. Features Extraction Method of Calf Scene-Interactive Behaviors

**Figure 4:**
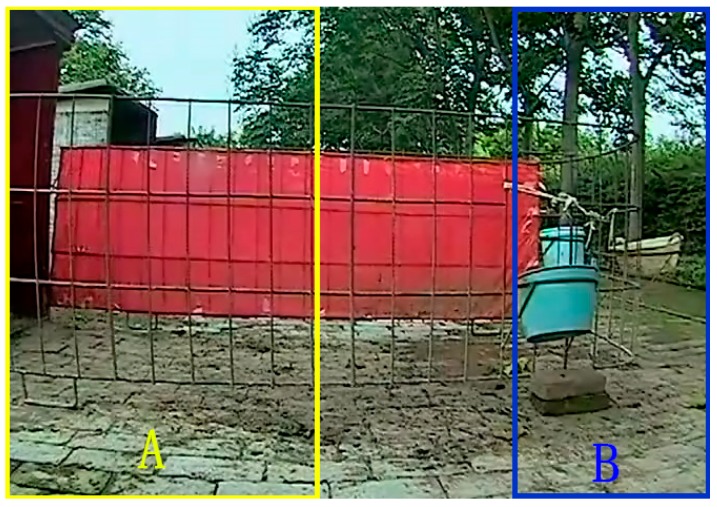
Calf scene-interactive behavior detection area. Calf’s behaviors of entering and leaving resting area were detected in Area **A** (yellow box: horizontal distance is body length of calf; resting area located on left of **A** zone). Feeding and drinking behaviors were detected in Area **B** (blue box: left border is left border of feeding-basin).

The calf entering or leaving the resting area was recorded on the left of the side-video view. When the right border of the target was in Area A (yellow box in Figure 4), the behavior was defined as entering or leaving the resting area. Feeding and drinking behaviors occurred on the right of the side-video view. When the target’s right border reached Area B (blue box in Figure 4), the front video was acquired, the feeding-basin and drinking-basin areas were extracted, and feeding and drinking behaviors were tested.

For extracting entering or leaving behaviors in resting area, the motion characteristics of the individual calf were combined to establish the following behavior recognition model. As animal behavior was continuous, the characteristic average of 10 consecutive frames was taken as the last feature, as shown in Equations (2)–(6)

(2) $$\left\{ \begin{array}{c} \overset{n+5}{\underset{i=n-5}{\sum }}\frac{B_{R}\left(i\right)}{10}-\overset{n+10}{\underset{i=n}{\sum }}\frac{B_{R}\left(i\right)}{10}>10,\;n\geq 6\\ \overset{n+5}{\underset{i=n-5}{\sum }}\frac{B_{D}\left(i\right)}{10}-\overset{n+10}{\underset{i=n}{\sum }}\frac{B_{D}\left(i\right)}{10}>10,\;n\geq 6\\ |\overset{n+5}{\underset{i=n-5}{\sum }}\frac{B_{L}\left(i\right)}{10}-\overset{n+10}{\underset{i=n}{\sum }}\frac{B_{L}\left(i\right)}{10}|<30,\;n\geq 6\\ B_{L}\left(i\right)<30,\;i=n-5 \end{array}\right.$$

(3) $$\left\{ \begin{array}{c} |\overset{n+5}{\underset{i=n-5}{\sum }}\frac{B_{R}\left(i\right)}{10}-\overset{n+10}{\underset{i=n}{\sum }}\frac{B_{R}\left(i\right)}{10}|<3,\;n\geq 6\\ |\overset{n+5}{\underset{i=n-5}{\sum }}\frac{B_{D}\left(i\right)}{10}-\overset{n+10}{\underset{i=n}{\sum }}\frac{B_{D}\left(i\right)}{10}|<3,\;n\geq 6\\ |\overset{n+5}{\underset{i=n-5}{\sum }}\frac{B_{L}\left(i\right)}{10}-\overset{n+10}{\underset{i=n}{\sum }}\frac{B_{L}\left(i\right)}{10}|<3,\;n\geq 6 \end{array}\right.$$

(4) $$\left\{ \begin{array}{c} \overset{n+10}{\underset{i=n}{\sum }}\frac{B_{R}\left(i\right)}{10}-\overset{n+5}{\underset{i=n-5}{\sum }}\frac{B_{R}\left(i\right)}{10}>10,\;n\geq 6\\ \overset{n+10}{\underset{i=n}{\sum }}\frac{B_{D}\left(i\right)}{10}-\overset{n+5}{\underset{i=n-5}{\sum }}\frac{B_{D}\left(i\right)}{10}>10,\;n\geq 6\\ |\overset{n+5}{\underset{i=n-5}{\sum }}\frac{B_{L}\left(i\right)}{10}-\overset{n+10}{\underset{i=n}{\sum }}\frac{B_{L}\left(i\right)}{10}|<30,\;n\geq 6\\ B_{L}\left(i\right)<30,\;i=n-5 \end{array}\right.$$

(5) $$\left\{ \begin{array}{c} \{\begin{array}{c} |\overset{n+5}{\underset{i=n-5}{\sum }}\frac{B_{R}\left(i\right)}{10}-\overset{n+10}{\underset{i=n}{\sum }}\frac{B_{R}\left(i\right)}{10}|<3,\;n\geq 6\\ \overset{n+5}{\underset{i=n-5}{\sum }}\frac{B_{D}\left(i\right)}{10}-\overset{n+10}{\underset{i=n}{\sum }}\frac{B_{D}\left(i\right)}{10}>10,\;n\geq 6\\ \overset{n+10}{\underset{i=n}{\sum }}\frac{B_{L}\left(i\right)}{10}-\overset{n+5}{\underset{i=n-5}{\sum }}\frac{B_{L}\left(i\right)}{10}>10,\;n\geq 6 \end{array}\\ or\\ \{\begin{array}{c} \overset{n+10}{\underset{i=n}{\sum }}\frac{B_{R}\left(i\right)}{10}-\overset{n+5}{\underset{i=n-5}{\sum }}\frac{B_{R}\left(i\right)}{10}>10,\;n\geq 6\\ \overset{n+10}{\underset{i=n}{\sum }}\frac{B_{D}\left(i\right)}{10}-\overset{n+5}{\underset{i=n-5}{\sum }}\frac{B_{D}\left(i\right)}{10}>10,\;n\geq 6\\ |\overset{n+10}{\underset{i=n}{\sum }}\frac{B_{L}\left(i\right)}{10}-\overset{n+5}{\underset{i=n-5}{\sum }}\frac{B_{L}\left(i\right)}{10}|<3,\;n\geq 6 \end{array}\\ and\\ B_{L}\left(i\right)<30,\;i=n-5 \end{array}\right.$$

(6) $$\left\{ \begin{array}{c} \{\begin{array}{c} \overset{n+5}{\underset{i=n-5}{\sum }}\frac{B_{R}\left(i\right)}{10}-\overset{n+10}{\underset{i=n}{\sum }}\frac{B_{R}\left(i\right)}{10}>10,\;n\geq 6\\ \overset{n+5}{\underset{i=n-5}{\sum }}\frac{B_{D}\left(i\right)}{10}-\overset{n+10}{\underset{i=n}{\sum }}\frac{B_{D}\left(i\right)}{10}>10,\;n\geq 6\\ |\overset{n+10}{\underset{i=n}{\sum }}\frac{B_{L}\left(i\right)}{10}-\overset{n+5}{\underset{i=n-5}{\sum }}\frac{B_{L}\left(i\right)}{10}|<3,\;n\geq 6 \end{array}\\ or\\ \{\begin{array}{c} |\overset{n+5}{\underset{i=n-5}{\sum }}\frac{B_{R}\left(i\right)}{10}-\overset{n+10}{\underset{i=n}{\sum }}\frac{B_{R}\left(i\right)}{10}|<3,\;n\geq 6\\ \overset{n+10}{\underset{i=n}{\sum }}\frac{B_{D}\left(i\right)}{10}-\overset{n+5}{\underset{i=n-5}{\sum }}\frac{B_{D}\left(i\right)}{10}>10,\;n\geq 6\\ \overset{n+5}{\underset{i=n-5}{\sum }}\frac{B_{L}\left(i\right)}{10}-\overset{n+10}{\underset{i=n}{\sum }}\frac{B_{L}\left(i\right)}{10}|>3,\;n\geq 6 \end{array}\\ and\\ B_{L}\left(i\right)<30,\;i=n-5 \end{array}\right.$$

where $B_{R}\left(i\right)$ is the right border of the target area in the *i*-th frame, $B_{D}\left(i\right)$ is the distance between the left and right borders of the target area in the *i*-th frame, and $B_{L}\left(i\right)$ is the left border of the target area in the *i*-th frame.

As the calf’s resting area was dark and the calf was black and white, the black parts of the calf that overlapped with the resting area could be lost during target detection when the calf entered or left the resting area. Therefore, we figured out the bias and considered that the calf started to enter or leave the resting area when *B_L_*(*i*) < 30 pixels. Besides this, we experimentally determined that the moving boundary before and after changed by more than 10 pixels when the calf entered, left the resting area, or turned around. The border fluctuation range was less than three pixels when the calf was stationary.

The calf was considered to enter the resting area if three features of the target area satisfied Equation (2). When Equation (3) was satisfied, the calf would be considered as stationary. The calf was considered to leave the resting area when Equation (4) was satisfied. When Equations (5) or (6) was satisfied, the calf would be considered as turning around.

### 2.4. Feeding and Drinking Behaviors Monitoring and Analysis

Background subtraction in the grayscale image was used to detect if there was a calf in the feeding/drinking area. If no calf was present, the current frame would be taken as a new background frame to continue the detection until the calf appeared. During this period, median filtering was used to pre-process data, and Otsu’s method was used for segmentation [31]. When the calf was eating, the head extended into the feeding basin. The bottom border of the acquired target area corresponded to the bottom border of the basin mouth, and the target had a larger area. The bottom border of the basin was denoted as *D_f_*, the bottom border of the target area was *D_t_*, and the area of the target was *S*. Considering the variability in the boundary of the target area, a threshold value of *D_f_* − 5 was used in the test. After the experiment, the area threshold was set as 1950 pixels.

(7) $$\{\begin{array}{c} D_{t}\geq D_{f}-5,\;D_{f}=48\\ S\geq 1950 \end{array}$$

where *D_f_* is the bottom border of the basin; *D_t_* is the bottom border of the target area; and *S* is the proportion of the target area. When the detection area satisfied Equation (7), the calf was considered to be feeding. Otherwise, it was considered as not feeding.

## 3. Results and Discussion

### 3.1. Target Detection Results

**Figure 5:**
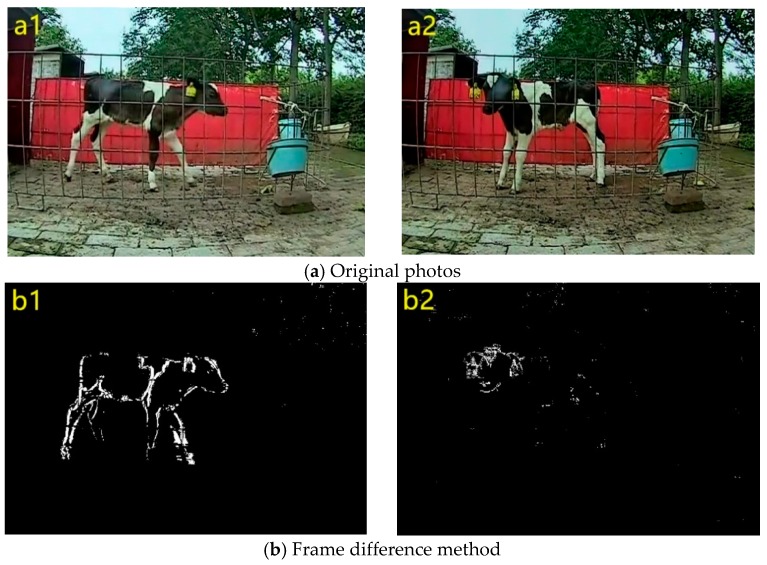

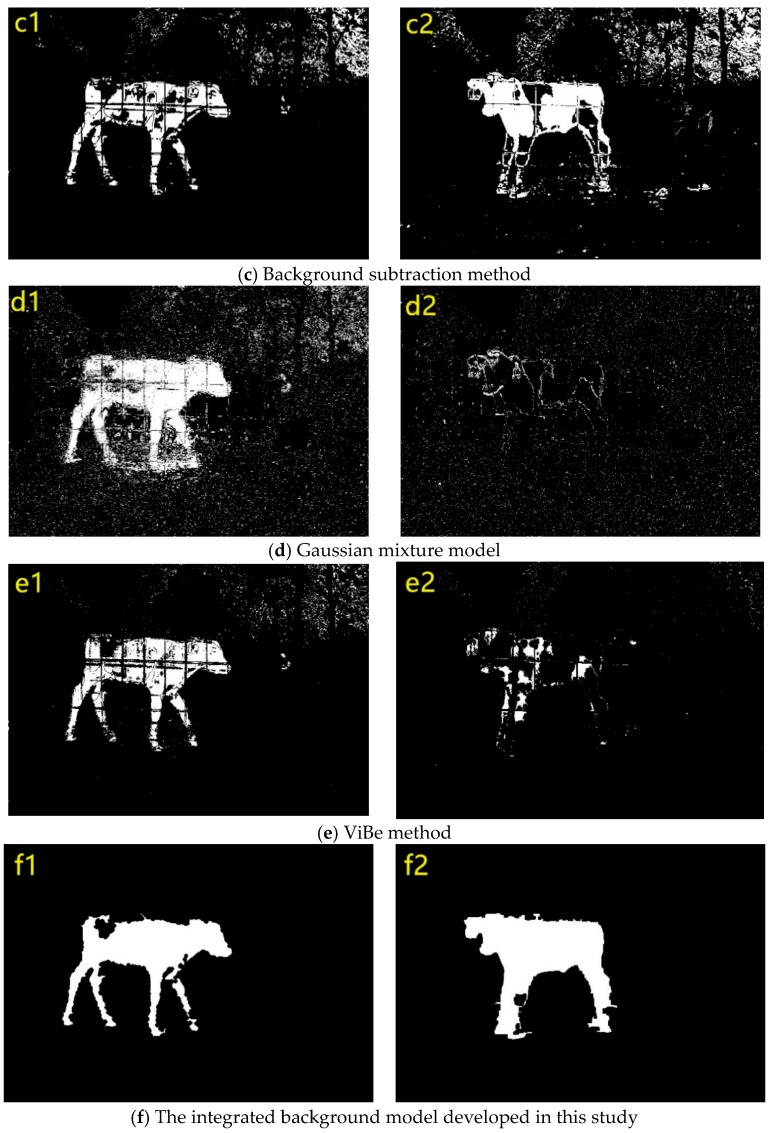
Dairy calf detection with different methods. The left column shows the detection results of different methods of calves in motion. The right column is the detection results of different methods of calf in stationary. **b**–**f** corresponds to the detection results of the frame difference method, background subtraction method, gaussian mixture model, ViBe, and the method of this paper.

The selected videos that included the calf in the resting and activity areas totaled 20,640 frames. The experiments were performed using the inter-frame difference method, the background subtraction method, the Gaussian mixture model, ViBe, and the new integrated background model developed in this study. The first column in Figure 5 shows detection of a calf in motion and the second column shows detection of the calf in stationary.

As shown in Figure 5b, the inter-frame difference method had strong noise rejection but it could not detect static and slow-moving targets. The conventional background subtraction method was able to detect most areas of dynamic and static targets but exhibited noise and poor adaptability. The Gaussian mixture model and ViBe had better noise immunity and detected dynamic targets, but were still unable to detect targets with continuous or small-amplitude motions. The new method of the integrated background model included the advantages of the inter-frame difference and conventional background subtraction methods, i.e., strong noise resistance and adaptability, and clearly detected most areas of dynamic and static targets.

### 3.2. Recognition of Entering/Leaving Behaviors in Resting Area

**Figure 6:**
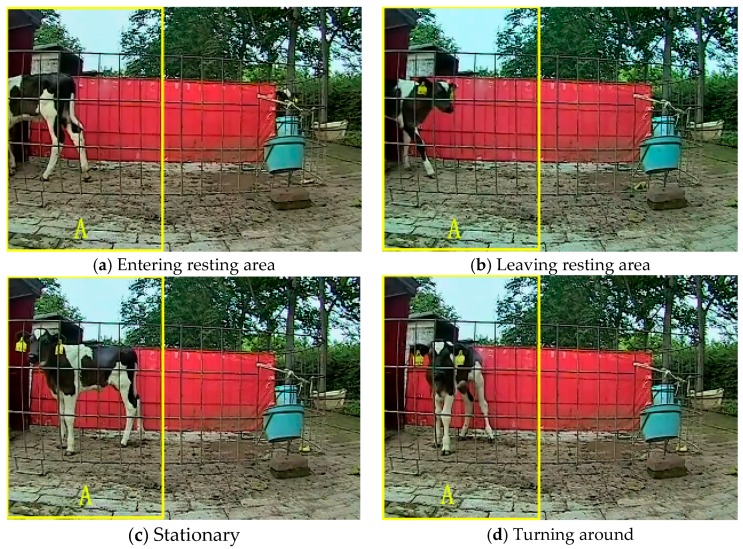
Example behaviors in Area A: (**a**) the calf is entering the resting area; (**b**) the calf is leaving the resting area; (**c**) calf is stationary; and (**d**) calf is turning around.

**Figure 7:**
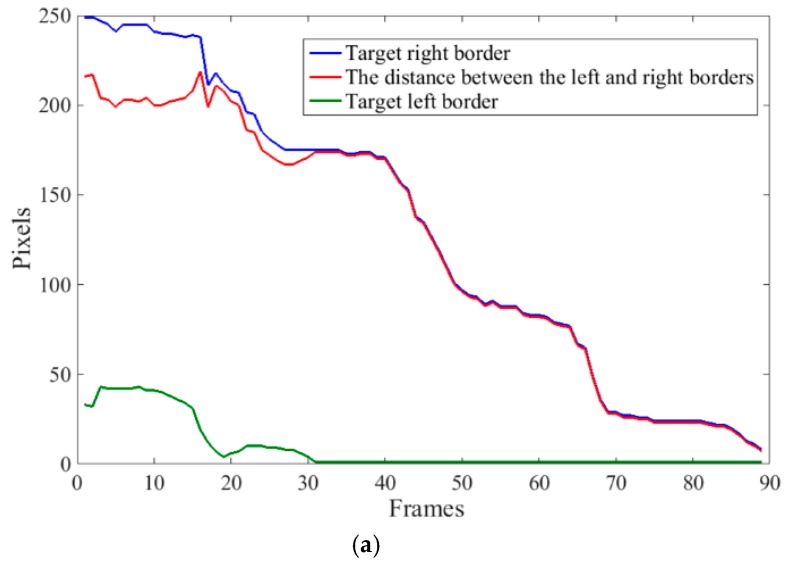

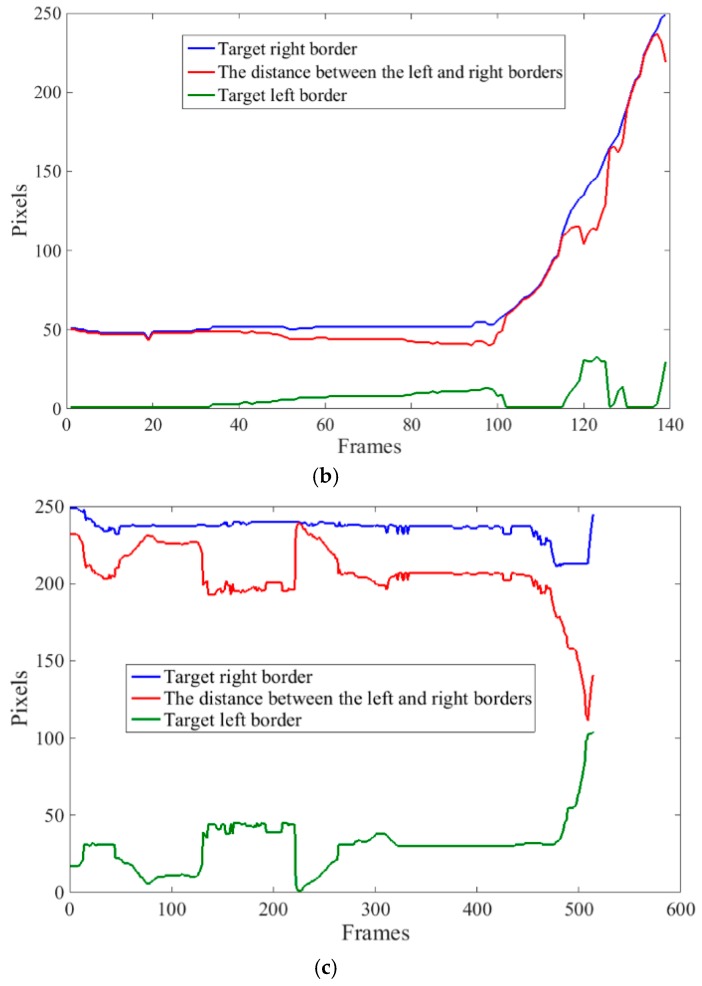
Extraction of behavioral characteristics: (**a**) entering resting area; (**b**) stationary and leaving resting area; and (**c**) stationary and turning around.

When the right border of the target was in Area A in Figure 6, identification of the calf entering or leaving the resting area was performed. Monitored Area A was the inlet and outlet of calf’s resting area. Monitored behaviors in Area A include entering the resting area, leaving the resting area, stationary (not moving), and turning around. The right border, left border, and the distance between the two borders were used as classification features. Figure 6 shows four behavioral examples. The extracted characteristic curves are shown in Figure 7.

As shown in Figure 7a, when the calf was approaching the resting area, the right border and the distance between the target’s right and left borders started to decrease, as well as the left border. When the calf was entering the resting area, the left border was essentially unchanged. In Figure 7b, the calf was static in the first 102 frames, where the first three features were more or less unchanged. When the calf was leaving the resting area, the right border and the distance between the target’s right and left borders started to increase. In Figure 6c, the head of the calf was facing the resting area. In the first 480 frames, the target’s right border was unchanged. The left border and the distance between the left and right borders suddenly changed because of a slight twisting of the front half of the calf. After the first 480 frames, the right border started to decrease, then became stable, and finally increased again. The distance between the left and right borders gradually decreased, then increased, and finally the left border gradually increased as the calf turned around.

The video segments containing the behaviors of entering the resting area, leaving the resting area, static behavior, and turning around had a total of 42,950 frames. The recognition rate (as compared to video review manually) are shown in Table 1.

The recognition rates of calf’s entering and leaving behaviors in the inlet and outlet of the resting area, stationary, and turning around were 94.38%, 92.86%, 96.85%, and 93.51%, respectively (Table 1). Failures in recognizing the entering or leaving behaviors were due to their being dark in color and the calf being black and white. The area of the calf that overlapped with the resting area could be missed during target detection. In this study, we used the average of 10 consecutive frames to calculate the characteristic value for recognizing behaviors, so static behavior was occasionally misjudged as entering or leaving the resting area when the calf entered or left the resting area from the static state/stationary. Besides, head swinging also led to misjudgment of the static behavior as turning around. In turning around, detected left and right border information sometimes remained essentially unchanged, with both the forelimbs and hindlimbs moving, resulting in misjudgment or detection.

### 3.3. Feeding and Drinking Behaviors Identification

**Figure 8:**
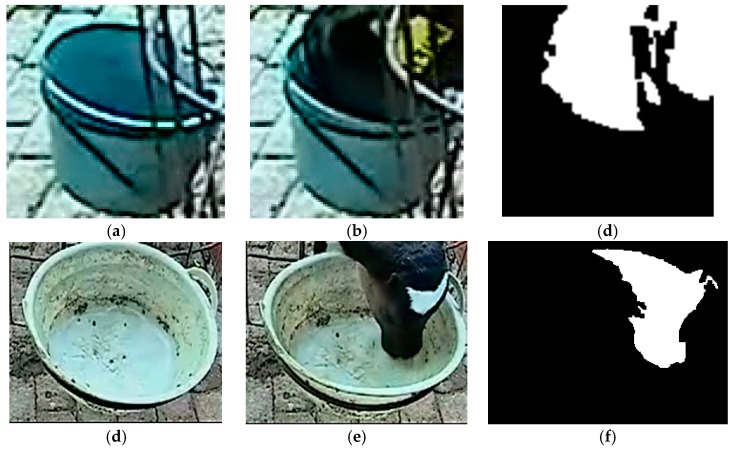
Feeding-basin and drinking-basin areas. (**a**) Feeding basin; (**b**) Calf feeding; (**c**) Result of binary image acquisition; (**d**) Drinking basin; (**e**) Calf drinking; (**f**) Result of binary image acquisition.

When the target’s right border reached the feeding/drinking area, the front video could be acquired. Based on the front video, the feeding-basin area (91 × 91 pixels) and drinking-basin area (251 × 192 pixels) were extracted (Figure 8). A square 4 × 4 pixel element was selected for the closing operation, extraction of the maximum area, and hole filling (Figure 8c,f).

**Figure 9:**
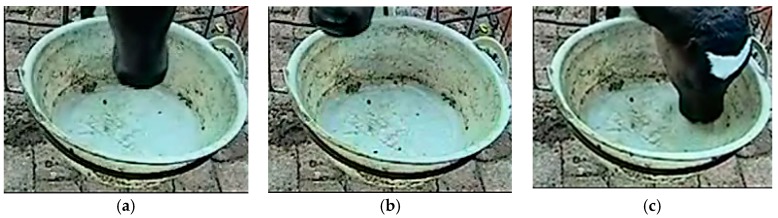
Target behavior in drinking area. (**a**) Preparing for drinking; (**b**) Looking around; (**c**) Drinking.

During drinking, the calf’s head area accounted for a large proportion of the field of view. When the calf’s head had just entered the basin, it was not in the drinking state and accounted for a smaller proportion of the view (Figure 9a). In addition, ‘looking’ behavior occurred in the drinking area (Figure 9b), but occupied a small area. In this study, drinking and non-drinking behaviors were distinguished by setting the detected area threshold of *S_t_* = 2900 pixels. When the proportion of the detected area was greater than *S_t_*, it was considered to be drinking behavior, otherwise it was considered to be non-drinking behavior.

In total, 1080 frames were sampled in the feeding area and 2045 frames in the drinking area. When the calf’s head was detected in these areas, the characteristics of the target area were extracted and used to identify whether the calf was feeding or drinking. The recognition accuracy was estimated in as: TP/(TP + TN), where TP is the number of samples that were correctly identified and TN is the number of samples that were erroneously identified; for feeding and drinking behaviors, these were 79.69% and 81.73%, respectively.

Problems with the recognition of feeding behavior could occur if the calf’s head was stationary in the feeding-basin before and after eating, or if the shadow of the calf’s head was mistakenly recognized as a feeding behavior. Feeding is a continuous process and it was difficult to separate pre- and post-feeding behavior from feeding behavior during the study. Licking the basin edge and the smelling the basin resulted in failures in the identification of drinking behaviors. Besides, only one calf was used to test the newly developed method, because commercial dairy farms usually put only one calf in a pen. As some farms may put a number of calves in a large pen, future studies will be required to optimize the method for the behavior-tracking of individual and groups of calves on commercial dairy farms.

In this study, only the daytime video was recorded. In the future, we will further develop the algorithm to recognize the behavior of calves at night. Besides animal behavior monitoring with 2D cameras, other non-invasive/remote monitoring technologies (e.g., heart rate monitor and infrared thermal) can also be added to the existing system to expend the functions or increase the accuracy of the dairy calf behavior monitoring system.

## 4. Conclusions

In this study, a new method (i.e., Integrated Background Model) was built by combining background-subtraction and inter-frame difference methods to monitor the behaviors of the dairy calf. By using the new model and motion characteristics of the calf in different areas of the enclosure, we successfully identified the behaviors of entering the resting area (94.38%), leaving the resting area (92.86%), remaining stationary (96.85%), turning around (96.85), feeding (79.69%), and drinking (81.73%).

The new method was tested with satisfied detection performance such as anti-interference characteristics for both dynamic and static targets, as compared to inter-frame difference and the background subtraction methods, Gaussian Mixture Model, and ViBe model. This newly developed method provides a basis for inventing evaluation tools to monitor calves’ health and welfare on dairy farms.

## Figures and Tables

**Table 1:** Results of calf’s behaviors recognition rate (%).

Actual Behavior	Classification Results				
	Entering the Resting Area	Leaving the Resting Area	Still	Turning	Missed Detection
(1) Entering the resting area	94.38	-	-	-	5.62
(2) Leaving the resting area	-	92.86	-	-	7.14
(3) Static behavior	0.43	0.57	96.85	1.43	0.72
(4) Turning around	-	-	5.56	93.51	0.93

Note: The recognition rate refers to the ratio of the number of correctly identified frames to the total number of frames in a behavior sample, and the ratio of the number of misclassified frames to the total number of frames in the behavior.
